# Ivabradine toxicity: a case report

**DOI:** 10.1186/s13256-022-03554-w

**Published:** 2022-10-24

**Authors:** Kavisha Singh, Muthukumar R. Alagarraju, Carl E. Wolf, Justin L. Poklis, Nitin Kulkarni, William Tharpe, Parag H. Joshi

**Affiliations:** 1 Division of Cardiology, Department of Internal Medicine, University of Texas Southwestern Medical Center, 5323 Harry Hines Blvd., Dallas, TX 75390-8830 USA; 2 Virginia Commonwealth University, Richmond, VA USA

**Keywords:** Arrhythmias, Pharmacokinetics, Cardiology, Case report

## Abstract

**Background:**

We describe a case of symptomatic bradycardia resulting from ivabradine toxicity by measurement of ivabradine levels, of which there are limited reports in the literature.

**Case presentation:**

A 43-year-old White female presented with several days of near syncope and dizziness accompanied by a drop in her heart rate to 50 beats per minute. She was taking ivabradine for inappropriate sinus tachycardia. After excluding several other causes of bradycardia, we made the diagnosis of ivabradine toxicity by measurement of serum ivabradine levels, an approach that is currently not clinically available.

**Conclusions:**

Measurement of serum ivabradine levels and knowledge of the pharmacokinetic properties of the drug can be utilized to confirm the diagnosis of ivabradine toxicity.

**Supplementary Information:**

The online version contains supplementary material available at https://doi.org/10.1186/s13256-022-03554-w.

## Background

Ivabradine inhibits the *I*_f_ current, which results in a pure negative chronotropic effect without any effect on inotropy. It is approved by the Food and Drug Administration (FDA) for use in symptomatic heart failure with reduced ejection fraction when patients have a persistent heart rate > 70 beats per minute (bpm); however, it is also used off label for treatment of inappropriate sinus tachycardia [1]. There are only a handful of cases in the literature describing ivabradine toxicity, usually resulting in bradycardia, and only two of them measure ivabradine levels [2–4]. In a trial of 3260 patients examining ivabradine and outcomes in heart failure, the incidence of bradycardia was 10%; therefore, this is a fairly common side effect of ivabradine [5]. Ivabradine use is contraindicated in patients with sick sinus syndrome, sinoatrial block, or third-degree AV block [6]. Our case reports adds to the cases of ivabradine toxicity or overdose and establishes the diagnosis by finding higher-than-expected serum ivabradine levels.

## Case presentation

**Fig. 1:**
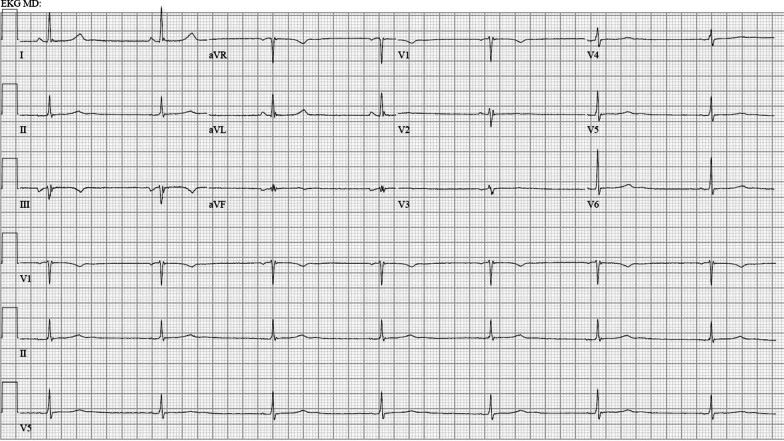
Patient’s initial rhythm on presentation to the hospital

**Fig. 2:**
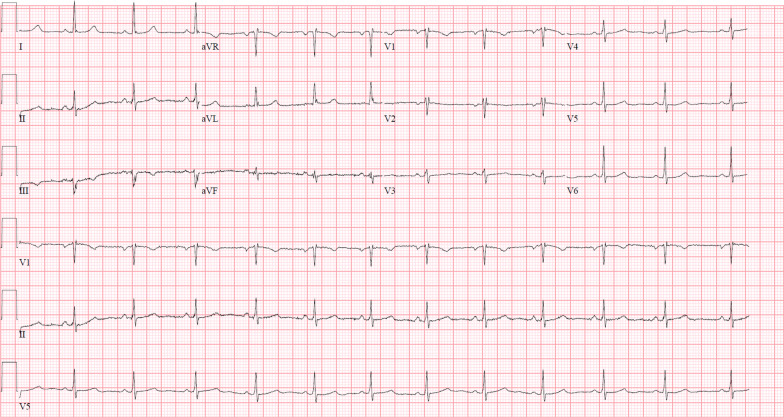
Patient’s baseline electrocardiogram

A 43-year-old White female presented with several days of dizziness culminating in a near-syncopal episode. She took multiple medications, including ivabradine 5 mg twice daily for symptomatic inappropriate sinus tachycardia, with a baseline heart rate around 110 bpm. After noticing a recent drop in her HR to 50 bpm, she discontinued her ivabradine 10 days prior to presentation. Her heart rate remained in the 50–55 bpm range prior to presentation. On arrival, she was afebrile, HR was 55 bpm, BP was 92/55 mmHg, and her oxygen saturation was 97% on room air. Orthostatic vitals were normal, and her physical examination was unremarkable other than a bradycardic pulse with regular rate. Her electrocardiogram (ECG) (Fig. 1) demonstrated an ectopic atrial rhythm with HR of 41 bpm (PR interval 120 milliseconds, QRS 80 milliseconds, QTc 397 milliseconds) with overnight telemetry showing sinus arrest and intermittent junctional rhythm. Her baseline ECG demonstrating sinus rhythm is shown in Fig. 2.

### Past medical history

She had a complex medical history including a qualitative platelet dysfunction disorder with multiple deep vein thromboses and pulmonary emboli on apixaban, granulosa cystic ovarian tumor, right nephrectomy for pyelonephritis, chronic pancreatitis, adrenal insufficiency, and hypertension. She worked in the pharmaceutical industry and did not consume tobacco, alcohol, or drugs. Her medication list included albuterol inhaler, apixaban, cetirizine, cholecalciferol, clonazepam, cyanocobalamin spray, eszoplicone, fludrocortisone, hydrocortisone, ivabradine, sertraline, potassium, and magnesium (Additional file 1).

### Differential diagnosis

The differential diagnosis for a patient with bradycardia is wide. The first determination in a patient with bradycardia is whether it is the result of conduction abnormalities in the sinus node or the atrioventricular (AV) system [7]. In the presented case, the normal PR interval and the absence of dropped beats suggested no AV conduction disease. Sinus bradycardia can be a manifestation of various intrinsic or extrinsic etiologies [7, 8]. Medications that can exacerbate sinus bradycardia include antihypertensive, anti-arrhythmic, and psychoactive drugs [8].

### Investigations

Given the acute presentation, an acquired cause was suspected. Thyroid-stimulating hormone (TSH) (3.51 μIU/mL), AM cortisol (13.3 μg/dL), serum creatinine (1.2 mg/dL), potassium (4.0 mmol/L), and magnesium (1.9 mg/dL) were normal. A recent echocardiogram showed normal ejection fraction and no major structural abnormalities. There were no signs of ischemia or heart failure (troponin T < 0.01 ng/mL, N-terminal pro BNP 64 pg/mL) and no evidence of situational syncope or carotid sinus hypersensitivity. After ruling out common intrinsic and extrinsic causes, ivabradine toxicity stood out as a potential cause of her bradycardia. Considering drug interactions, we found no agents on her medication list that exacerbate the effects of ivabradine [1].

### Management

**Fig. 3:**
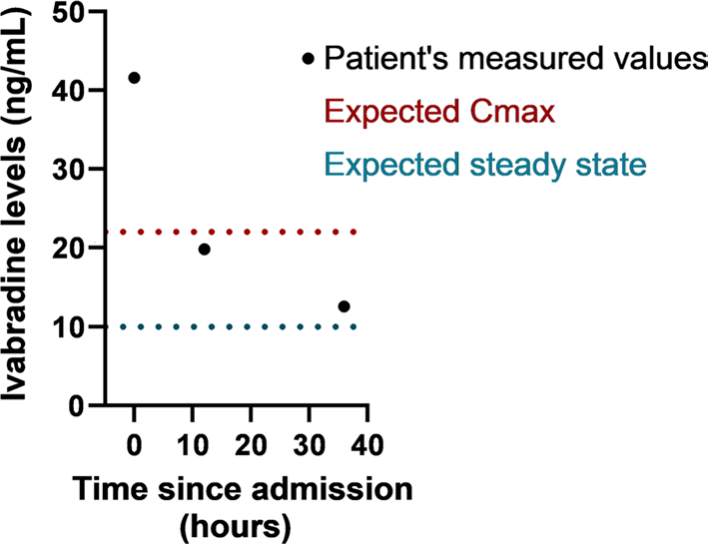
Expected versus observed ivabradine levels in our patient

Given her symptoms, ivabradine was withheld and she was discharged from the hospital after 2 days of observation in stable condition with discontinuation of ivabradine. Her heart rate upon ambulation was 70 bpm on discharge. Serum specimens were sent to the Toxicology Laboratory at Virginia Commonwealth University Medical Center for analysis as measurement of ivabradine levels could not be performed at our institution. Ivabradine concentrations at admission, 12 hours and 36 hours post-admission were 42 ng/mL, 20 ng/mL, and 13 ng/mL, respectively. Ivabradine is expected to follow near-linear elimination with a half-life of 6 hours per the manufacturer, while clinical measurements estimate it at 11 hours [6]. With chronic administration at 5 mg twice daily, the maximum plasma concentration (*C*_max_) expected is approximately 22 ng/mL 1 hour after oral administration and the mean steady-state plasma concentration is 10 ng/mL (Fig. 3) [9]. Given the time of the last dose, the serum ivabradine levels should have been undetectable, but instead were twice the expected *C*_max_. Therefore, we concluded that her bradycardia was secondary to ivabradine toxicity.

## Discussion and conclusions

Sinus node disease can result from a variety of causes (Table 1), but ivabradine toxicity should be considered in patients on this medication. Given the primary action of ivabradine on the *I*_f_ current, which depresses cardiac automaticity, drug toxicity can also manifest as visual disturbances in the settings of polypharmacy [1, 10].

**Table 1:** Common causes of sinus node dysfunction

Drugs (beta blockers, lithium, calcium channel blockers, digoxin, clonidine, amiodarone, propafenone, parasympathomimetic drugs)
Myocardial infarction
Severe hypoxia
Hypothyroidism
Hypothermia
Fibrodegenerative changes of sinus node
Vomiting
Vasovagal syncope
Carotid sinus stimulation
Raised intracranial pressure

Sinus node dysfunction can range from sinus bradycardia, SA node exit block or sinus arrhythmia to sinus pause or arrest [11]. Making the diagnosis via ECG alone can be challenging since the mass of the SA node is too small to create an electrical signal on a surface ECG. The P wave seen on ECG is a reflection of atrial tissue depolarization rather than sinus node depolarization.

First-degree SA nodal exit block results from a fixed delay in the conduction of SA node to surrounding atrial tissue with preserved 1:1 conduction between the two. This delay cannot be seen on a surface ECG.

Second-degree SA nodal exit block can be type I (Wenckebach) or type II. Type I second-degree SA nodal block has shortening P–P intervals prior to a dropped P wave, which results in a pause less than two P–P cycles due to increasing sinoatrial conduction times in smaller increments [12]. In type II second-degree SA nodal exit block, dropped P waves at regular intervals result in pauses that are a multiple of the normal baseline P-P interval.

In third-degree SA nodal exit block, electrical activity in the SA node is present but cannot conduct to the surrounding atrial tissue. The surface ECG will show no sinus node P waves, and an escape rhythm may be present. Pauses will have a P–P interval in multiples of the baseline P–P interval similar to type II second-degree SA nodal exit block.

In sinus arrest, there is complete absence of sinus node activity with no P wave with no recordable sinus node electrogram on invasive study. On ECG, sinus arrest and third-degree SA nodal exit block may look identical. In sinus arrest, the length of the pause on the ECG is not a multiple of the baseline sinus cycle length.

Our patient presented with hemodynamically stable bradycardia due to an ectopic atrial rhythm, which could be due to depressed automaticity from blockade of *I*_f_ current by ivabradine resulting in a depressed sinus node and takeover by an ectopic atrial pacemaker [13]. There has been a suggestion of this in previous reports with prolonged sinoatrial conduction time on electrophysiology studies [14]. This delay cannot be seen on a surface ECG. In other prior reports of ivabradine toxicity, sinus bradycardia has been the predominant rhythm with heart rates as low as 31 bpm with drug level 525 ng/mL and heart rate 50 bpm with drug level 375 ng/mL [2, 4]. The expected HR reduction with ivabradine is 10 bpm at recommended doses. Our patient’s heart rate dropped significantly below her baseline; however, her mild bradycardia was likely a function of having only two to three times the expected serum drug levels and having a healthy escape rhythm.

In this case, detectable and higher-than-expected serum ivabradine concentrations were confirmed with multiple samples. This raises the possibility that there was an error in the time of reported last dose or that she was a slow metabolizer. Ivabradine is metabolized by the liver and intestines via CYP3A4 oxidation. Creatinine clearance above 15 mL/minute and mild-to-moderate hepatic impairment have minimal impact on ivabradine clearance and should not have impacted serum levels in our patient [6]. Drug interactions should be considered when prescribing this medication since sertraline and clonazepam have also been associated with bradycardia [1, 15]. Given that the observed half-life of drug clearance in the hospital for our patient was 11 hours, which is within the range of previously reported clinical measurements, we concluded that the most likely cause of ivabradine toxicity was an error in her last reported dose. Sertraline is a CYP3A4 inhibitor and could have impacted ivabradine drug levels as well as half-life.

Ivabradine was permanently discontinued, and follow-up in clinic demonstrated normal sinus rhythm at 75 bpm. The patient has had no recurrence of arrhythmias or near syncope.

Sinus node dysfunction can have a variety of extrinsic and intrinsic causes. Varying degrees of sinus node dysfunction can be differentiated with closer evaluation of surface ECG but may require an invasive EP study. We present a case of ivabradine toxicity with bradycardia with an ectopic atrial rhythm and junctional escape rhythms, which to our knowledge is only the third case in the medical literature to make the diagnosis by ruling out several other potential causes of bradycardia and confirming detectable serum ivabradine levels with the help of specialized lab testing. This case reviews the pharmacokinetics of ivabradine and how we can use that knowledge to confirm ivabradine toxicity.

## Supplementary Information

**Additional file 1.** Complete drug list.

## Data Availability

The datasets generated and/or analyzed during the current study are not publicly available since individual patient data at our institution are protected by the Health Insurance Portability and Accountability Act.

## Abbreviations

AV: Atrioventricular
BNP: Brain natriuretic peptide
BP: Blood pressure
bpm: Beats per minute
ECG: Electrocardiogram
EP: Electrophysiology
HR: Heart rate
SA: Sinoatrial
TSH: Thyroid-stimulating hormone
