# Supplementary material for: Ivabradine toxicity: a case report
Source: J Med Case Rep. 2022 Oct 24;16:392. doi: 10.1186/s13256-022-03554-w (PMC9590231; doi:10.1186/s13256-022-03554-w)
Supplement: Supplementary file 1 — Additional file 1. Complete drug list. [file 13256_2022_3554_MOESM1_ESM.docx]

| ALBUTEROL SULFATE (VENTOLIN INHL) | as needed. |
| --- | --- |
| apixaban (ELIQUIS) 2.5 mg oral tablet | Take 2.5 mg by mouth twice daily. |
| AZASITE 1 % Opht ophthalmic drop |  |
| benzonatate (TESSALON PERLES) 100 mg oral capsule | Take 1 Cap (100 mg total) by mouth three times daily as needed for Cough. Do not open or chew capsules. |
| cetirizine (ZYRTEC) 10 mg oral capsule | Take 10 mg by mouth as needed. |
| cholecalciferol, Vitamin D3, (VITAMIN D3) 1,000 unit chewable tablet | Take 1,000 Units by mouth daily. |
| clonazePAM (KLONOPIN) 0.5 mg oral tablet | Take 0.5 mg by mouth as needed. |
| cyanocobalamin (NASCOBAL) 500 mcg/spray nasal spray | 1 Spray every Monday. |
| eszopiclone (LUNESTA) 3 mg oral tablet | Take 3 mg by mouth every other day. |
| fludrocortisone (FLORINEF) 0.1 mg oral tablet | Take 0.05 mg by mouth every other day. |
| hydrocortisone (CORTEF) 5 mg oral tablet | Take 2.5 mg by mouth twice daily. Reasons5mg w/breakfast, 5mg w/lunch, 2.5 w/dinner |
| ivabradine (CORLANOR) 5 mg oral tablet | 5 mg daily. |
| magnesium 250 mg oral tablet | Take 250 mg by mouth daily. |
| ondansetron (ZOFRAN) 8 mg oral tablet | Take 4 mg by mouth every 8 hours as needed. |
| PAZEO 0.7 % Opht Drop |  |
| polyethylene glycol 3350 (MIRALAX) 17 gram oral powder packet | Take 17 g by mouth daily as needed for Constipation. Mix in 8 oz. of fluid. |
| potassium bicarbonate (KLOR-CON/EF) 25 mEq effervescent oral tablet | Take 25 mEq by mouth daily. Dissolve in 4-8 oz. of water. |
| potassium chloride ER (MICRO-K) 10 mEq SR oral capsule | Take 10 mEq by mouth daily. Do not crush or chew. |
| prochlorperazine (COMPAZINE) 10 mg oral tablet | Take 10 mg by mouth three times daily as needed for Nausea. |
| promethazine (PHENERGAN) 25 mg oral tablet | Take 25 mg by mouth every 6 hours as needed for Nausea. |
| sertraline (ZOLOFT) 25 mg oral tablet | Take 3 Tabs (75 mg total) by mouth daily. |
| SOD PHOS,M-B/K PHOS,MONOB (K-PHOS MF ORAL) | Take 1 Tab by mouth daily. |
